# Molecular confirmation of HIV-1 and HIV-2 coinfections among initially serologically dually-reactive samples from patients living in West Africa

**DOI:** 10.1371/journal.pone.0283602

**Published:** 2023-03-31

**Authors:** Boris K. Tchounga, Mélanie Bertine, Florence Damond, Valentine Marie Ferré, André Inwoley, Simon P. Boni, Alice Moisan, Jean-Christophe Plantier, Diane Descamps, Didier K. Ekouevi, Charlotte Charpentier

**Affiliations:** 1 Programme PACCI, Site ANRS, Abidjan, Côte d’Ivoire; 2 Elizabeth Glaser Pediatric AIDS Foundation, Yaoundé, Cameroun; 3 Service de Virologie, Université Paris Cité, INSERM, IAME, UMR 1137, AP-HP, Hôpital Bichat-Claude Bernard, Paris, France; 4 Félix Houphouet-Boigny University, Abidjan, Côte d’Ivoire; 5 Research and Diagnosis Center for AIDS and Other Infectious Diseases (CeDReS), CHU (University Hospital) of Treichville, Abidjan, Côte d’Ivoire; 6 Normandie Univ., UNIROUEN, EA2656, GRAM, CHU de Rouen, Laboratoire de Virologie associé au CNR du VIH, Rouen, France; 7 ISPED, Université de Bordeaux & Centre INSERM U1219—Bordeaux Population Health, Bordeaux, France; 8 Université de Lomé, Faculté des Sciences de la Santé, Département de Santé Publique, Lomé, Togo; 9 Centre Africain de Recherche en Epidémiologie et en Santé Publique (CARESP), Lomé, Togo; Institut Pasteur, FRANCE

## Abstract

**Objectives:**

This study aimed to confirm the co-infection with HIV-1 and HIV-2, among West African patients using in-house HIV type/group enzyme-immuno assays and molecular diagnosis.

**Design:**

A cross-sectional survey was conducted from April 2016 to October 2017 in the biggest HIV clinics of Côte d’Ivoire and Burkina Faso.

**Method:**

A first serological confirmation was done in the referral laboratory using an in-house, indirect immuno-enzymatic essay allowing the qualitative detection of both HIV-1 and HIV-2 antibodies. In order to separately detect anti-HIV-1 and anti-HIV-2 antibodies, a type/group specific enzyme-immuno assay (HIV-GSEIA) was used. To confirm the co-infections, HIV-1 and HIV-2 DNA-qualitative PCR assays were performed.

**Results:**

A total of 91 patients were enrolled in the study and provided blood sample for HIV type confirmatory testing including 13 (14.3%) HIV-2 mono-reactive and 78 (85.7%) HIV-1/HIV-2 dually-reactive based on the HIV testing National Algorithms. The first serological ELISA confirmatory test performed showed that 80 (78.9%) of the 91 participants were dually-reactive. The HIV-GSEIA performed on these 80 serum samples retrieve one 61 HIV-1/HIV-2 dually-reactive samples. HIV-1 and HIV-2 DNA PCR were performed on 54 of the 61 HIV-1/HIV-2 dually-reactive samples and 46 out of 61 (75.4%) samples were found HIV-1/HIV-2 coinfected.

**Conclusion:**

The contribution of type/group specific enzyme-immuno assay to accurately identify HIV-1/HIV-2 coinfections remain suboptimal, emphasizing the need for molecular diagnosis platforms in West Africa, to avail HIV DNA PCR test for the confirmation of HIV-1/HIV-2 co-infections.

## Introduction

West Africa is characterized by the co-circulation of HIV-1 and HIV-2, leading to co-infections with both viruses [1]. ART-naïve patients co-infected with both viruses were reported to experience a higher mortality rates compared to HIV-2 mono-infected patients [2, 3]. Moreover, providing long-term adequate antiretroviral (ARV) treatment to patients infected with both viruses is challenging in sub-Saharan African countries, where access to ARV drugs effective on patients living with HIV-2 and experiencing viral resistance to protease inhibitors (PI) and integrase inhibitors is still scarce [1, 4, 5].

The accurate diagnosis of this co-infection remains challenging for the national HIV testing algorithms of West African countries [6–8]. Currently, only molecular diagnosis methods consisting in the detection of HIV-1 and HIV-2 DNA in the same patients constitute a gold standard for the confirmation of HIV-1 and HIV-2 co-infections [6, 8]. Only one study described a cohort of 17 patients co-infected, with positive HIV-1 RNA and HIV-2 proviral DNA [9]. For most of the studies with no molecular diagnosis, there is persistent doubts on the accuracy of this diagnosis and the most appropriate expression to describe these patients is dually-reactive for HIV-1 and HIV-2.

The main challenge faced by the West African countries is access to molecular technics for the accurate diagnosis of HIV-1 and HIV-2 co-infections. Some in-house molecular tests have been developed, validated and patented for the molecular diagnosis of both HIV-1 and HIV-2. However these in-house or manufactured tests are scarcely available for referral laboratories of West African countries [8]. The alternative remains the systematic confirmation of HIV-1 and HIV-2 dually-reactive results from rapid tests used in the national algorithm, with a more specific and sensitive in-house ELISA technic that can be affordable and routinely used in referral laboratories.

The aim of this study was to confirm the co-infection with HIV-1 and HIV-2, among West African patients serologically dually-reactive based on the national HIV testing algorithms, using an in-house type/group ELISA assays and the molecular diagnosis assays.

## Methods

### Study design

A cross-sectional survey was conducted from April 2016 to October 2017 in the biggest HIV clinics of Côte d’Ivoire and Burkina Faso. During the study period, all adult patients classified as HIV-2 or HIV-1/ HIV-2 dually-reactive based on national testing algorithms, and who visited the facility for routine follow-up visit were invited to participate. After giving a written consent and the administration of a questionnaire, two 5 ml EDTA tube of blood were collected from each participant and sent for the HIV confirmatory test, to the referral laboratory of the study in Côte d’Ivoire.

### Biological procedures

A first serological confirmation was done in the referral laboratory using an in-house, indirect immuno-enzymatic test allowing the qualitative detection of both HIV-1 and HIV-2 antibodies. This test was adapted from an assay developed for HIV-1 and has shown good concordance with DNA PCR [10, 11]. It is routinely used in the reference laboratory of the study in Abidjan as home-made ELISA [8, 10]. Based on the results of this confirmation, samples with dual-reactivity were sent for further investigation to Bichat-Claude Bernard Virology laboratory in France.

In order to separately detect anti-HIV-1 and anti-HIV-2 antibodies, a type/group specific ELISA assay previously described was used [12]. Briefly, this manual ELISA assay combines, in a single run, the principles of both an indirect ELISA to study the binding of antibodies present in a given sample to peptides representing consensus sequences of HIV-1 groups M and O, and HIV-2 (type/group specific ELISA, GSEIA), and a subtype-specific enzyme immunoassay (SSEIA) [12]. All dually-reactive samples were sent to the virology laboratory of Rouen hospital where a primate Lentivirus Immuno Assay (PLIA) previously described was performed [13].

To confirm dual infections, HIV DNA qualitative PCR was performed. Total DNA was extracted from PBMC using QIAsymphony DSP DNA Mini Kit (Qiagen, Courtaboeuf, France). HIV-1 total DNA from PBMC was measured using a real-time PCR assay targeting a conserved consensus region in the Long Terminal Repeat region (GENERIC HIV DNA Cell, Biocentric^®^, Bandol, France) with a limit of quantification of 10 copies/PCR. HIV-2 total DNA detection was performed using in-house real time PCR assay previously described with a limit of quantification of 6 copies/PCR [14].

### Data analysis and statistical considerations

For this study we did not estimate a minimum sample size. However, according to the literature, for exploratory studies a minimum number of 30 patients is recommended [15, 16].

Data analysis was conducted using STATA version 14.0, (Stata Corp, College Station, Texas USA). Quantitative characteristics were presented as median with interquartile range, and qualitative data were presented as frequency with percentages.

### Ethical considerations

This study was part of the IeDEA West Africa HIV-2 cohort study approved by the National Ethic Committee for life Science and Health (CNESVS: IORG00075).

## Results

### Patients’ characteristics

A total of 91 patients were enrolled in the study and provided blood sample for HIV type confirmatory testing including 13 (14.3%) HIV-2 mono-reactive and 78 (85.7%) HIV-1/HIV-2 dually-reactive. The median age of the 91 participants was 53 years-old (IQR = 48–58), 55 (60.4%) of them were women, 86 (94.5%) were receiving ARV treatment and the median CD4 cell count at sampling was 481/mm^3^ (IQR = 336–604) (Table 1).

**Table 1:** Demographic characteristics of participants according to initial HIV status based on national HIV testing algorithms in West Africa.

	HIV status according to national testing algorithms					
	HIV-2		HIV-1&2		Total	
	n = 13 (14.3%)		n = 78 (85.7%)		n = 91	
**Age (years)**						
Median (IQR*)	54 (50–58)		53 (47–57)		53 (48–58)	
**Gender**						
**Female, n (%)**	4 (30.8)		51 (65.4)		55 (60.4)	
**Duration of HIV infection (years)**						
**Median (IQR)**	5.0 (0–7)		5.5 (3–10)		5.0 (3–10)	
**Country, n (%)**						
**Burkina Faso**	0 (0.0)		13 (16.7)		13 (14.3)	
**Cote d’Ivoire**	13 (100.0)		65 (83.3)		78 (85.7)	
CD4 (cells/mm^3^) at sampling						
≤350, n (%)	4 (30.8)		21 (26.9)		25 (27.5)	
**>350, n (%)**	9 (69.2)		57 (73.1)		66 (72.5)	
ART** initiation						
**Yes, n (%)**	13 (100.0)		73 (93.6)		86 (94.5)	

*IQR: Interquartile range

**ART: Antiretroviral therapy

### Result of the serological confirmation

**Fig 1:**
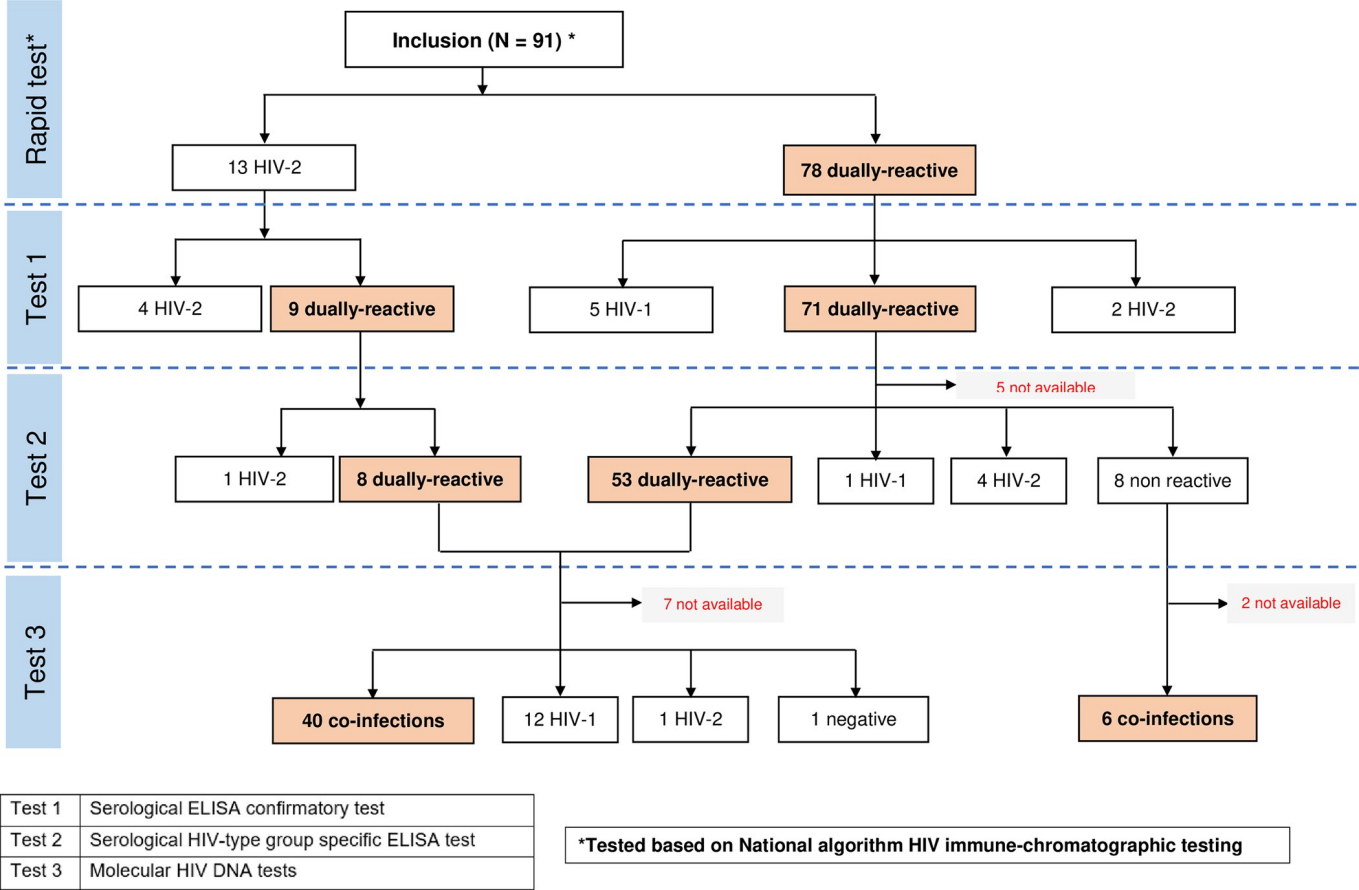
HIV serological and molecular testing for dually HIV-1 and HIV-2 diagnosis in West Africa.

The serological confirmatory test, performed in the referral laboratory in Côte d’Ivoire, showed that 71 (91.0%) of the 78 samples initially found dually-reactive were confirmed dually-reactive. Off the seven remaining samples five were HIV-1 mono-reactive and two were HIV-2 mono-reactive. Among the 13 samples initially identified HIV-2 mono-reactive, 4 (30.8%) were confirmed HIV-2 mono-reactive and the 9 remaining were found dually-reactive (Fig 1). Overall, 80 (78.9%) of the 91 participants were confirmed dually-reactive, while five were HIV-1 mono-reactive and six HIV-2 mono-reactive.

Among the 80 serum samples showing a dual-reactivity with the serological confirmatory test performed in Côte d’Ivoire, 75 could be assessed with a Group/type-Specific Enzyme ImmunoAssay (HIV-GSEIA). The results of HIV-GSEIA were as follows: 61 HIV-1/HIV-2 dually-reactive samples, five HIV-2 mono-reactive, one HIV-1 mono-reactive, and eight non-reactive samples. An additional type-specific serological technique, PLIA assay, could be performed for a subset of samples with available plasma. Thus, 54 of the 61 dually-reactive samples with the HIV-GSEIA could be assessed with the PLIA assay, and all were found to be dually-reactive with the PLIA assay (Fig 1).

### HIV molecular specific assays

HIV-1 DNA and HIV-2 DNA PCR were performed on 54 of the 61 HIV-1/HIV-2 dually-reactive samples according to the HIV-GSEIA test. A co-infection with both positive HIV-1 DNA and HIV-2 DNA PCR was evidenced in 40 samples. The remaining samples showed 12 HIV-1 mono-infections, 1 HIV-2 mono-infection and one sample was negative by both HIV-1 and HIV-2 DNA PCR. Among the eight non-reactive samples with the HIV-GSEIA test, HIV-1 DNA and HIV-2 DNA PCR could be performed for six of them, all six showing dual-infections (Fig 1). Overall, a total of 46 out of 61 (75.4%) patients were found HIV-1/HIV-2 coinfected. Among the 9 dually-reactive sample from the 13 HIV-2 initially diagnosed and reclassified as HIV-1 and HIV-2 dually-reactive, all of them were confirmed coinfected with HIV-1 and HIV-2 using DNA testing.

## Discussion

In this study, 91 patients initially HIV-2 mono-reactive or HIV-1/HIV-2 dually-reactive using national algorithm for HIV testing were included. The use of ELISA HIV type-specific tests followed by HIV-1 and HIV-2 DNA PCR tests led to formally identify 46 (50.5%) HIV-1/HIV-2 co-infected patients among the 91 participants.

### Conformation of low accuracy of rapid tests

This study confirmed the previously reported challenges faced by the national HIV testing algorithms of most West African countries to accurately diagnosed HIV-1/HIV-2 co-infection. Thus, a previous diagnosis confirmatory study conducted in three West African countries, reported that only 23% of patients being dually-reactive for HIV-1 and HIV-2 with national testing algorithms where confirmed dually-reactive with a more sensitive ELISA test, while 40% of them were reclassified as mono-infected with HIV-1 [8]. Additional studies trying to identify an algorithm based on improved-version of rapid tests, also reported good diagnosis performances for HIV-2 mono-reactive samples, but failed to identify a strong algorithm for dually-reactive samples [6, 7].

### Contribution and limitation of immunochromatographic tests

In the present study, the confirmatory ELISA test confirmed 71 out of the 78 dually-reactive samples initially detected by the immuno-chromatographic rapid test, and revealed that nine of the 13 samples initially detected as HIV-2 mono-reactive were actually dually-reactive samples. Thus, the use of an ELISA test increases the reliability in discriminating between mono-reactive and HIV-1/HIV-2 dually-reactive samples. Finally, 76.7% of the dually-reactive samples by serological tests were confirmed as co-infection by HIV DNA PCR, suggesting that the use of an ELISA group specific test, already implemented in West Africa, among HIV-2 and HIV-1/HIV-2 dually-reactive samples could be a first step to increase reliability of HIV type diagnosis, but remain insufficient for accurate diagnosis. In addition, we found a high proportion of HIV-1 and HIV-2 mono-infections among patients initially showing serological profile of HIV-2 or HIV-1/HIV-2 dually-reactive. This confirms the limit of an initial rapid immuno-chromatographic test to correctly discriminate HIV-2 mono-infections and HIV-1/HIV-2 co-infections and emphasize the need to rapidly deploy HIV DNA PCR testing at least in referral laboratories of each West African countries, to confirm the diagnosis of HIV-2 and HIV-1/HIV-2 co-infections [8, 14].

### Public health perspectives

The low diagnostic accuracy of national testing algorithms using rapid tests, has impaired the management of ARV drugs in West African countries for a long period, as a quarter of dually-reactive patients were misclassified HIV-1 mono-infected patients receiving PI-based regimen as preferred first-line ART [5, 17]. This problem became less harmful since the implementation of the 2019 WHO guidelines with the integrase inhibitor dolutegravir-based regimen as recommended first-line regimen for all HIV patients [18]. However, the challenge of long-term management of HIV-2 and HIV-1/HIV-2 co-infected patients remains, as many studies reported resistance mutations to integrase inhibitors and to protease inhibitors that could be used as alternative in case of virological failure [4]. It is therefore critical to reinforce the diagnosis of the co-infection with both viruses, especially in West African countries, where treatment optimization after occurrence of virological failure with resistance is still an important challenge for HIV-2 and HIV-1/HIV-2 co-infected patients [17, 19]. In addition, it would be appropriate to document the immunological and virological response of truly co-infected patients compared to HIV-1 or HIV-2 infected patients.

### Study limitations

This study aimed to perform a molecular confirmation of the HIV-1/HIV-2 co-infection in patients living in West Africa and diagnosed as dually-reactive with immune-chromatographic rapid test. The confirmation tests were done sequentially, using only the previously confirmed samples for the next step. This approach did not allow the calculation of concordance coefficient and the comparison of different ELISA assays with the different algorithms of each country using DNA PCR testing as gold standard. However, the molecular confirmation of the HIV-1/HIV-2 co-infected patients performed in this study will lead to the description of the biggest cohort of co-infected patients in West Africa with the perspective of addressing some specific question of interest around long-term clinical and immuno-virological response, immunologic and inflammatory phenomena related to disease progression, as well as possible viral competition [1, 9].

### Conclusion

Despite the contribution of type/group specific enzyme-immuno assay to accurately identify HIV-/HIV-2 coinfections, there is a critical need for implementation of molecular diagnosis platforms in West Africa, to avail HIV DNA PCR test as gold standard for the confirmation of HIV-1/HIV-2 co-infections.
